# Targeted generative data augmentation for automatic metastases detection from free-text radiology reports

**DOI:** 10.3389/frai.2025.1513674

**Published:** 2025-02-06

**Authors:** Maede Ashofteh Barabadi, Xiaodan Zhu, Wai Yip Chan, Amber L. Simpson, Richard K. G. Do

**Affiliations:** ^1^Ingenuity Labs Research Institute, Department of Electrical and Computer Engineering, Queen's University, Kingston, ON, Canada; ^2^School of Computing and Department of Biomedical and Molecular Sciences, Queen's University, Kingston, ON, Canada; ^3^Department of Radiology, Memorial Sloan Kettering Cancer Center, New York, NY, United States

**Keywords:** synthetic data generation, targeted data augmentation, metastases detection, natural language processing, large language models, free-text radiology report

## Abstract

Automatic identification of metastatic sites in cancer patients from electronic health records is a challenging yet crucial task with significant implications for diagnosis and treatment. In this study, we demonstrate how advancements in natural language processing, namely the instruction-following capability of recent large language models and extensive model pretraining, made it possible to automate metastases detection from radiology reports texts with a limited amount of gold-labeled data. Specifically, we prompt Llama3, an open-source instruction-tuned large language model, to generate synthetic training data to expand our limited labeled data and adapt BERT, a small pretrained language model, to the task. We further investigate three targeted data augmentation techniques which selectively expand the original training samples, leading to comparable or superior performance compared to vanilla data augmentation, in most cases, while being substantially more computationally efficient. In our experiments, data augmentation improved the average F1-score by 2.3, 3.5, and 3.9 points for lung, liver, and adrenal glands, the organs for which we had access to expert-annotated data. This observation suggests that Llama3, which has not been specifically tailored to this task or clinical data in general, can generate high-quality synthetic data through paraphrasing in the clinical context. We also compare metastasis identification accuracy between models utilizing institutionally standardized reports vs. non-structured reports, which complicate the extraction of relevant information, and show how including patient history with a customized model architecture narrows the gap between those two setups from 7.3 to 4.5 points on F1-score under LoRA tuning. Our work delivers a broadly applicable solution with remarkable performance that does not require model customization for each institution, making large-scale, low-cost spatio-temporal cancer progression pattern extraction possible.

## 1 Introduction

Cancer is the second leading cause of death globally, responsible for one in six deaths.^1^ Most cancer-related fatalities are due to metastatic diseases (Dillekås et al., 2019), which can be partly prevented through early diagnoses. Discovering spatio-temporal patterns of cancer progression and their responsiveness to individual factors can guide healthcare providers toward more targeted monitoring for metastases, enabling timely interventions (Sherman et al., 2016). In contrast to traditional clinical studies that demand long-term trials and extensive investment, we are interested in a novel data-driven approach for tracking cancer progression that leverages the available electronic health records (EHR), specifically the routine computed tomography (CT) scans in cancer patients. In this context, our study proposes a natural language processing (NLP) enabled solution for systematically identifying metastatic sites from radiology reports. Our work not only facilitates the extraction of spatial and temporal patterns in cancer spread but also enables the comparison of cancer progression under different circumstances, significantly enhancing our understanding of metastatic tropism and improving patient outcomes.

Despite the abundance of raw clinical data, the availability of labeled datasets is very limited due to cumbersome annotation processes and data privacy concerns, which hinder data sharing between healthcare institutions and research groups. The data we experimented on were collected at Memorial Sloan Kettering Cancer Center (MSKCC) over a span of 10 years, and clinical experts have annotated small subsets of data to indicate the presence or absence of metastases in different organs, namely lung, liver, and adrenal glands. We leverage the impressive in-context capability of instruction-tuned large language models (LLMs) to expand our labeled dataset. We instruct the LLM to generate synthetic data and train a small language model (SLM) on the expanded dataset to detect metastases. Our approach effectively mitigates data scarcity without requiring extra manual annotation.

**Figure 1:**
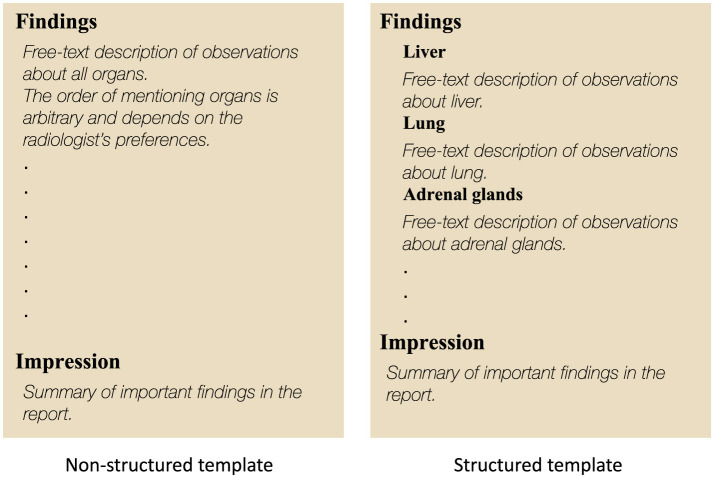
Illustration of structured and non-structured report templates. The non-structured template is a broadly adopted report style among radiologists. Structured template refers to the institutional template designed by radiology departments for their own internal use.

The radiology reports in our data follow the institutional standardized structured template, illustrated in Figure 1, which organizes the findings section into 13 subsections that discuss individual organs or organ groups separately. Previous studies (Batch et al., 2022; Do et al., 2021) utilized this report template to detect metastases in each organ using the corresponding subsection and showed promising performance. Our approach, however, leverages only the impression section of the report, a standard element of radiology reports that summarizes key observations, enabling broader adaptability across different report structures and bypassing variation of institution-level templates. However, there is a trade-off here as the impression-only setting falls short compared to the structured report setting in terms of performance, which we address partially by proper model design and data curation. Overall, we leverage the emergent ability of open-source LLMs in paraphrasing clinical text to enhance the metastases identification from the impression section and rigorously evaluate the effect of data augmentation (DA), patient history, and parameter-efficient fine-tuning (PEFT) techniques in addressing the performance gap inherent in the impression-only setting. Our main contributions are summarized as follows:

- Leveraging LLMs for DA: our work is one of the pioneering efforts to leverage open-source LLMs to generate synthetic data in the radiology report context. We introduce and assess targeted DA strategies that focus only on the most informative samples to enhance model performance.
- Proposing a generalized metastasis detection methodology: we conduct a systematic comparison of structured findings vs. impression-only settings, resulting in a more broadly applicable approach for metastasis detection. Additionally, by relying only on text modality, our method sidesteps the complexities associated with varying imaging techniques, which makes our method adaptable across institutions.
- Enhancing baseline performance: we utilize patients' prior radiology records, which substantially narrows down the performance gap between structured and impression-only report settings. We also demonstrate how PEFT methods, specifically prompt-tuning and LoRA, show superior performance compared to traditional fine-tuning on small training data.
- Conducting comprehensive evaluation: we perform an extensive evaluation of our methods on datasets annotated for metastases in the lung, liver, and adrenal glands, highlighting the practical benefits and limitations of our approach under different circumstances.

The remainder of this paper is structured as follows: Section 2 reviews the relevant literature, covering DA techniques in NLP, with a focus on generative DA applied to medical tasks, and provides an overview of popular PEFT methods. Section 3 describes the proposed solution, including data description, problem formulation, the augmentation process, the proposed model architecture, and its training procedure. Section 4 presents the experimental results, offering a thorough analysis of the effectiveness of augmentation techniques, the impact of report structures, and the role of prior reports on model performance using various PEFT methods. Section 5 features the discussion, providing insights into the diversity of generated examples and the influence of patient history duration in metastases analysis. Finally, Section 6 concludes the paper by summarizing the key findings and their clinical implications.

## 2 Related works

### 2.1 Data augmentation in natural language processing

DA is a widely used technique to enhance the performance of deep learning models, especially in low-data scenarios. It increases the size and diversifies the training data without requiring additional data collection and annotation. While various methods exist for generating synthetic samples, most approaches in classification tasks focus on creating samples similar to existing ones through label-preserving transformations. However, these transformations are challenging to define due to the discrete and complex nature of language, where even a single word change can alter the meaning entirely. Traditional DA methods, such as easy data augmentation (EDA) (Wei and Zou, 2019), synonym replacement using predefined ontologies or Masked Language Models (Wu et al., 2019), and back-translation (Shleifer, 2019), often introduce label noise into the training data, disrupting original data distribution. Generative DAs are more powerful but used to be limited to conditional generative models that required training separate models per class (Anaby-Tavor et al., 2020).

Recently, the advent of instruction-tuned LLMs has revolutionized DA and enabled in-context generative DA, which produces lexically diverse yet semantically consistent samples without any task-specific training (Dai et al., 2023). Their deep language understanding and ability to follow human instructions closely are key to this advancement. Despite all the progress, the benefits of DA and the most effective DA techniques remain task-dependent (Chen et al., 2023; Piedboeuf and Langlais, 2023). In this work, we apply in-context generative augmentation on the metastatic sites identification task by paraphrasing the impression section of radiology reports. We use Llama3 (Dubey et al., 2024), a powerful open-source transformer-based LLM, to generate synthetic samples.

Various targeted augmentation techniques have been suggested in previous works to improve efficiency and downstream task accuracy compared to vanilla augmentation. Møller et al. (2024) conduct minority class augmentation to mitigate performance degradation resulting from class imbalance. Lin et al. (2023) keeps only the generated samples with high pointwise v-information, which provide significant informational value to the model. However, their method has demonstrated improvement in classification problems with many classes and may not be suitable for binary classification tasks. Additionally, their approach involves post-generation filtering of synthetic samples, which does not reduce the LLM generation workload. Sahu et al. (2023) generates challenging samples near class boundaries by prompting a LLM with two samples from different classes and asking the model to generate samples that are a mix of those classes and closer to a dominant class. Although their method is suitable for general domain tasks, where the LLM can gain a satisfactory understanding of each class by one provided example, we believe it is not suitable for complex domain-specific tasks. We later introduce three targeted augmentation methods that filter training samples pre-generation and have been designed considering the special properties of our dataset.

### 2.2 Generative data augmentation in medical domain

Several studies have demonstrated the benefits of LLM-based DA in the medical domain, particularly when data is scarce. Yang et al. (2023) applied generative DA with GPT family models to enhance the task of radiology report simplification. In their approach, they generated simplified versions of existing texts and paraphrased the original pairs to expand their expert-generated dataset. They report considerable improvements in performance when training BART model (Lewis et al., 2020) on the augmented data. Guo et al. (2023) utilized ChatGPT^2^ and GPT-4 (Achiam et al., 2023) to generate new medical question-answer pairs and rephrase the existing ones within the pubMedQA dataset. They train two SLMs on the augmented data and find that the effectiveness of augmentation depends on the specific SLM trained, the LLM used, and the augmentation technique, which varies significantly from substantial performance gain to a notable negative impact. Due to the sensitive nature of the data and privacy concerns, we do not use API-based models to generate new samples. We instead use LLama3 model, which showed comparable generation capabilities with API-based models. It is worth mentioning that the data has been anonymized, and the sensitive information has been removed or altered, complying with ethical and legal regulations. To the best of our knowledge, this work is the first to apply in-context generative DA to a classification task on radiology reports.

### 2.3 Parameter-efficient fine-tuning techniques

PEFT methods have steadily gained popularity as language models continue to grow in size since they reduce the memory footprint during training compared to full fine-tuning (FFT). While these methods were once thought to involve a trade-off between efficiency and performance, further research has shown that they outperform FFT in certain setups while updating < 1% of model parameters. PEFT methods are specifically designed for transformer-based language models and modify the transformer block by adding a small set of trainable parameters while freezing the remaining ones. Prompt-tuning prepends artificial trainable tokens to the keys and values in the attention layers, a core module of the transformer block. Among different prompt-tuning variations, we use P-tuning v2 (Liu et al., 2021), which is directly applicable to natural language understanding tasks and has shown comparable performance to FFT across different scales. LoRA (Hu et al., 2021), another popular PEFT method, introduces additive trainable rank decomposition matrices into linear layers inside the transformer block, which have the capability of merging into pretrained weights in the inference stage. Their experiments illustrate that LoRA is the most beneficial if only applied to the value and query transformation matrices. In this work, we utilize prompt-tuning and LoRA to adapt a SLM to the task of metastases detection from radiology reports.

## 3 Materials and research methods

### 3.1 Data description

The data used in this study were collected at MSKCC from July 2009 to May 2022 by waiver of informed consent and follows a structured departmental template, as shown in Figure 1. Subsets of the data have been annotated by five radiologists for the presence or absence of metastases in designated organs, namely lung, liver, and adrenal glands; for detailed information about the process of gathering and annotating data, please refer to Batch et al. (2022). All the necessary steps to ensure patient privacy have been taken; sensitive information about the patient's identity has been removed from the text, the patient identifiers have been replaced with randomly generated identifiers, and the dates of examination were altered by applying a random shift per patient.

Table 1 presents the details of the data split across the train, validation, and test sets for each organ, with the split performed at the patient level to prevent data leakage between training and testing. The size of the annotated data varies by organ, including 869 patients for the lung, 404 patients for the adrenal glands, and 315 patients for the liver. To account for this variability, we adjusted the train/validation/test split ratios to ensure that the test set remains sufficiently large and representative of the data diversity. For the lung, liver, and adrenal glands datasets, we allocated 15%, 50%, and 40% of patients to the test set, respectively. The validation set comprises 15%, 25%, and 25% of the patients for each organ, leaving 70%, 25%, and 35% of patients for training. With an average of approximately nine reports per patient, this results in 5,265 reports for the lung, 674 for the liver, and 1,506 for the adrenal glands in the training set. The rate of metastatic occurrence also differs by organ, with the liver showing the highest frequency at 31%, the lungs at 16%, and the adrenal glands at just 7%. This variation in data sizes and metastatic occurrence rates allows us to evaluate our method's performance under diverse conditions. Data preprocessing is the same as described in our previous work (Barabadi et al., 2024).

**Table 1:** Data split details for all organs.

	**Freq**	**Train**			**Validation**			**Test**		
		**Percentage**	**Subjects**	**Reports**	**Percentage**	**Subjects**	**Reports**	**Percentage**	**Subjects**	**Reports**
Lung	16%	70%	606	5265	15%	131	1,221	15%	130	1,179
Liver	31%	25%	78	674	25%	79	670	50%	158	1,433
Adrenal glands	7%	35%	145	1506	25%	97	919	40%	162	1,682

### 3.2 Problem formulation

We represent each patient in the dataset D with a tuple (R, Y^*^), where *R* = (*r*_1_, *r*_2_, ..., *r*_*l*_) denotes a list of the patient's radiology report texts in chronological order and $Y^{*}=\left(y_{1}^{*},y_{2}^{*},...,y_{l}^{*}\right)$ represents a list of corresponding binary labels. *l* is the total number of available reports for the patient, varying from 1 to 30 reports in our data. $y_{i}^{*}\in \left\{0,1\right\}$ indicates the presence or absence of metastatic disease in the organ at the time of report *r*_*i*_, serving as our ground-truth label. We apply vanilla or targeted DA method to *r*_*i*_, which prompts a LLM to generate a list of paraphrased versions of *r*_*i*_, *R*_*i, syn*_ with length 0 ≤ *l*_*i, syn*_. Then, we compose $D_{syn}$ by randomly matching reports from *R*_(*i* = 1*tol*), *syn*_ and generating *l*_*max, syn*_ synthetic patients, where $l_{max,syn}=\underset{1\leq i\leq l}{\max}l_{i,syn}$. This guarantees the maximum use of the generated synthetic reports and aligns our report-level data augmentation method with the patient-level model architecture. Finally, we combine the original and synthetic dataset to form the augmented dataset $D_{aug}=D+D_{syn}$. Our objective is to train a classifier $F\left({\left\{r_{i}\right\}}_{i=1}^{j}\right)$, which, given the patient history up to report *r*_*j*_, outputs *y*_*j*_, i.e. the predicted probability of metastatic disease at the time of *r*_*j*_. In the following subsections, we describe the different augmentation techniques we proposed and the design of classifier *F* and its training process.

### 3.3 Data augmentation process

**Figure 2:**
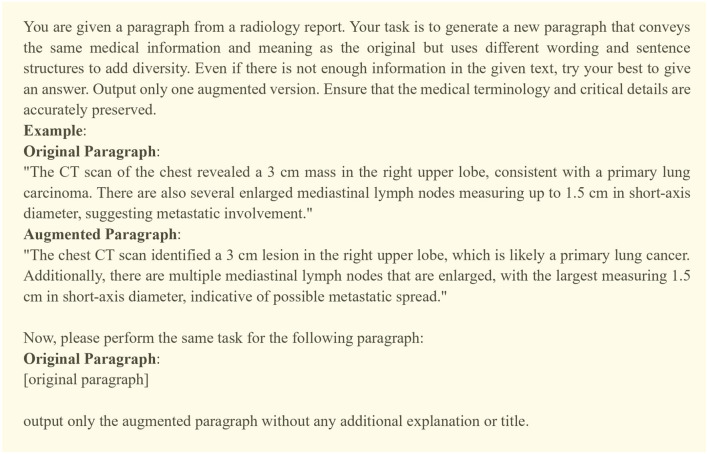
The constructed prompt for synthetic data generation. We incrementally revised the prompt by qualitatively evaluating LLM's outputs.

Our DA techniques involve prompting LLMs to paraphrase impression text samples. The prompt, as shown in Figure 2, includes a brief background, a task description, and a demonstration containing an original and paraphrased impression text, providing the model with a clear illustration of the task. We used ChatGPT to generate the example in the prompt and its rephrased version. We use the same prompt for all three organs and prompt the model multiple times to generate N variations of each original report in the training data. To ensure effective augmentation, it is essential to balance diversity and semantic faithfulness to the original data in the synthetic samples. Higher diversity enables the model to learn from varied expressions and terminologies, while semantic faithfulness ensures consistency with the original meaning, which is crucial for accurate learning. To quantify this, we evaluate the lexical diversity of the generated samples using the self-BLEU score (Zhu et al., 2018) and measure their semantic similarity to the original text through cosine similarity. These measurements, along with analysis of their implications, are presented in Section 5.1. We use LLama3-70B-instruct as the LLM in all the experiments, which has been pretrained and then instruction-tuned on a large amount of data. We set the repetition penalty to 1.15 and the temperature to 0.3 to encourage diverse yet meaningful outputs.

Besides vanilla in-context DA, which generates a fixed number of synthetic reports per an original report, we further examine three targeted augmentation methodologies, which, instead of naively treating all the training reports the same, select a subset of them to rephrase. It is clear that not every synthetically generated sample offers the same benefit to the model performance. This can be attributed to the fact that the LLM sometimes fails to generate a realistic example by preserving the label, or the generated example does not offer any new information to the SLM trained in the subsequent step. However, it is not trivial how to identify the most valuable synthetic samples to use during training. We are further interested in predicting the usefulness of synthetic samples before even generating them by assessing the original sample at hand. Therefore, we can avoid the generation cost for the samples that are going to be discarded. We use the insight we gained from the data to design some proxies to find a suitable subset of training samples to expand. We discuss each method and the rationale behind it in detail in the following paragraphs.

**Length filtering:** The intuition behind this method is that when the source sample is a short paragraph that does not contain much information, augmentation does not offer a considerable benefit since the LLM fails to rephrase the source sample in different ways and ends up outputting very similar versions to the original sample and each other. So, we filter the source samples based on the number of words and only generate samples similar to the ones that are longer than the threshold. Based on the text length distribution in the data, we set the threshold to a minimum of 20 words.

**K-fold misclassification-based filtering (KF-MF):** The idea behind this method is to identify the samples more susceptible to misclassification and generate synthetic data exclusively from those challenging samples to improve model performance. However, evaluating a model on its own train set leads to a very low error rate due to overfitting and does not help in finding challenging train samples. To mitigate this issue, we use a *k*-fold cross-validation strategy when we split the source samples into *k* chunks and pick one as the validation set and the rest as the train set in every step, which leads to training k separate models. The performance of each model on the unseen validation chunk is not under the effect of overfitting and can be utilized for detecting challenging samples. So, we aggregate the misclassified samples from all *k* chunks and only prompt the LLM to rephrase those samples. We set *k* = 5 and trained each model for 20 epochs before using it to detect misclassified samples.

**Minority class augmentation:** As discussed before, our dataset labels are highly imbalanced. We initially addressed this issue by upsampling the minority class. Here, we investigate another approach where we augment only the reports from the patients with positive labels for at least half of their reports, and we replicate those patients with a factor that approximately balances the number of samples in the positive and negative classes.

### 3.4 Model architecture

**Figure 3:**
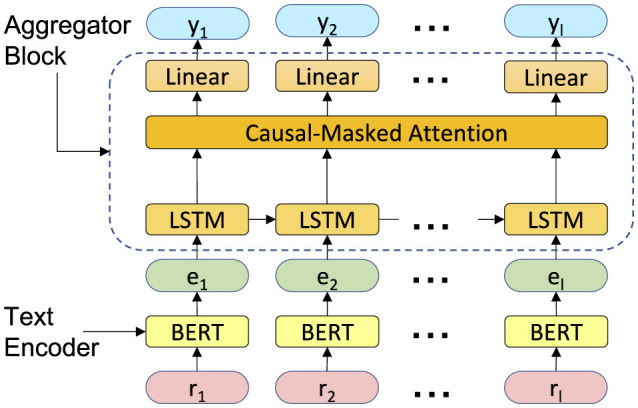
The proposed model architecture. It contains two main blocks: a text encoder and an aggregator block.

The classifier architecture has been shown in Figure 3. It consists of a text encoding block and an aggregator block. The text encoding block embeds the radiology texts into a customized semantic space and generates pre-report text embeddings. The aggregator block, which consists of a LSTM, an attention layer, and a classifier layer, links the information from patient history over time to determine the presence or absence of metastatic diseases. We explain the details of each block in the following paragraphs.

**Text encoding:** To encode information from the report text, represented in natural language, into a dense vector, we employ a pretrained SLM. Due to limited knowledge of SLMs in specific domains, such as medical terminology, using them immediately to encode clinical data would result in poor performance. We fine-tune the pretrained SLM on the metastatic site identification task, initializing from its pretrained weights, which allows the model to familiarize itself with the targeted domain while leveraging its prior knowledge. Various methods have been proposed for tuning transformer-based language models on downstream tasks. In this study, we employ vanilla fine-tuning, as well as two PEFT methods: prompt-tuning (Liu et al., 2021) and LoRA (Hu et al., 2021).

Prompt-tuning prepends trainable prompt tokens to the input of each transformer block and keeps the pretrained weights frozen. Prompt length is a hyperparameter that can control the number of tunable parameters, and its optimal value depends on the task at hand. As recommended by previous works (Liu et al., 2021), we initialize prompt tokens with the embedding of relevant words from the model vocabulary for better performance and training stability. LoRA, on the other hand, assumes that updating weights for a new task can be represented by a low-rank matrix. Therefore, rather than directly adjusting the original weights, the update is decomposed into the multiplication of a horizontal and vertical matrix. To prevent adding random noise to the pretrained weights at the beginning, one of these matrices is initialized to zero. The rank of the update matrix is a hyperparameter that controls the number of tunable parameters. Based on the original paper (Hu et al., 2021), the optimal combination of linear layers in the transformer block to update with LoRA is the query and value transformation matrices while keeping the rest of the parameters frozen.

We denote the text encoder model as M, where $e_{i}=M\left(r_{i}\right)$. Here, $e_{i}\in R^{d}$ represents the embedding for report *r*_*i*_, and *d* is the internal dimension of the SLM. We use BERT-base in our experiments, which has the hidden dimension *d* of 768.

**Aggregator block:** The aggregator block passes the text embedding of reports, $e_{i=1}^{l}$, through a LSTM layer followed by a multi-head attention layer and finally a linear classification layer. For computational efficiency, we engineered the aggregator block to compute *y*_*j*_ for all 1 ≤ *j* ≤ *l* simultaneously. We down-project *e*_*i*_s before feeding them to the LSTM layer, which lowers the dimension to 128. The LSTM layer then processes the down-projected text embeddings chronologically, updating its internal state at each timestep with new information, while the attention layer processes the information in parallel and takes relevant information from all timesteps equally into account. To ensure the aggregator block remains causal, i.e. *e*_*i*_ for *i*>*j* does not involve in the computation of *y*_*j*_, we use one-directional LSTM and a causal attention mask. Finally, we apply a linear classification head to the output of the attention layer at each timestep to generate the predicted labels, ${\left\{y_{i}\right\}}_{i=1}^{l}$.

### 3.5 Training process

We train the model by minimizing the binary cross-entropy loss function on the original data for a maximum of 1,000 epochs with early stopping on the validation set with 200 epochs of patience. For training on augmented data, we start from the model trained on the original data and continue the training for 10 epochs when *N* = 10. In the targeted augmentation experiments, we adjust the number of training epochs based on augmented data size to have the same computation budget during training as vanilla augmentation. We evaluate the model on the validation set during training after each epoch and pick the best model based on the validation F1-score. We use half-precision training and Adam optimizer to tune the weights with a cosine scheduler on the learning rate.

## 4 Results

We evaluate our proposed method against several baselines, each constructed by systematically removing key components—augmentation, patient history, and PEFT training—one at a time. This allows us to analyze their individual impact and gain deeper insights into their interactions. For the baselines that rely on a single report for prediction, we employ a simplified architecture comprising a SLM and a classification head. Additionally, We compare our method performance in structured and impression-only settings. In the structured report scenario, the input to the SLM, which was originally the impression text, is replaced by the organ-specific subsection combined with the impression text. Several of the baselines are adapted from our previous work (Barabadi et al., 2024), where they are discussed in detail. We rerun each experiment with five different random seeds and present both the average and the highest F1-scores achieved on the unseen test set. We discuss our main observations in the following sections.

### 4.1 Effectiveness of augmentation techniques

**Figure 4:**
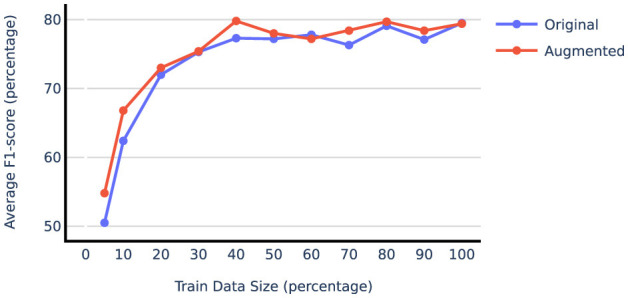
Average F1-score on lung test set when gradually increasing train size with and without data augmentation.

Table 2 compares the model performance when trained on the original train data vs. vanilla augmented data using both prompt-tuning and LoRA techniques. The results indicate that synthetic data generation significantly benefits the liver and adrenal glands datasets, while the performance on the lung dataset remains unchanged or declines when using the augmented data. This could be attributed to the fact that the lung dataset, with over 5,000 training report samples, is already sufficiently large, resulting in diminishing returns from additional samples. To test this hypothesis, we examined the effect of augmentation on subsets of the lung dataset of varying sizes. The results shown in Figure 4 illustrate that smaller subsets of data gain more F1-score improvement from data augmentation. Beyond 50% point, even adding real-world examples with gold labels yields minimal improvement. For subsequent augmentation experiments, we used 20% of the lung training data to compare different augmentation techniques. Table 2 also reveals that LoRA outperforms prompt-tuning, with LoRA also benefiting more from augmentation on average. Specifically, augmentation improved the average F1-score on the liver and adrenal glands test sets by 2.4 and 3.9 points when using LoRA, compared to 2.5 and 0.2 points when using prompt-tuning. More extensive experiments comparing PEFT methods are presented in Section 4.4.

**Table 2:** Data augmentation results on test sets.

	**Train data**	**PT**		**LoRA**	
		**Avg F1**	**Best F1**	**Avg F1**	**Best F1**
Lung	Original	76.4 ± 1.2	77.7	79.5 ± 1.5	81.4
	Augmented	74.7 ± 3	**78.2**	79.4 ± 0.6	80.1
Liver	Original	77.9 ± 2.5	80.8	82.6 ± 2	85.6
	Augmented	**80.4** ± 2.4	**83.2**	**85** ± 1.3	**86.6**
Adrenal glands	Original	67.1 ± 2.3	71.1	70.6 ± 6.4	79.7
	Augmented	**67.3** ± 5	**74.4**	**74.5** ± 3.6	77.4

Entries for Avg F1-score columns are in the format of average F1-score ± standard deviation of F1-score. PT stands for prompt-tuning. Bold values are the entries that show the augmented approach overperformed the original approach.

We compare targeted augmentation strategies in Table 3. First of all, DA always yields superior or comparable performance to training on original data in terms of the average F1-score. This is especially important considering that DA can result in performance degradation in some natural language processing tasks if applied without tailoring the augmentation process properly to the task at hand. For the lung dataset, all the targeted augmentation techniques show stronger performance compared to vanilla augmentation. In the liver dataset, KF-MF augmentation performs better compared to vanilla augmentation, which indicates the benefit of carefully identifying and repeating the most challenging samples for the model. Length filtering and minority class augmentation show a slight decline compared to vanilla augmentation on the liver dataset, but they are much more computationally efficient in terms of the number of generated samples and still outperform no augmentation baseline. For adrenal glands, length filtering performs the best between targeted DA methods, followed by KF-MF and minority class augmentation. Length filtering performs surprisingly well despite its simplicity. In general, the effectiveness of targeted DA methods is dependent on the dataset, and there is no unified trend in their performance compared to vanilla augmentation.

**Table 3:** Comparing vanilla augmentation with targeted augmentation strategies.

	**Metric**	**w/o DA**	**Vanilla DA**	**Length filtered DA**	**Minority class DA**	**KF-MF DA**
Lung	Avg F1	72 ± 2.7	73 ± 2.6	73.2 ± 1.2	73.6 ± 0.8	**74.3** ± 0.8
	Best F1	75.1	**75.9**	74.5	74.7	75.6
Liver	Avg F1	82.6 ± 2	85 ± 1.3	84.7 ± 2.5	84.2 ± 2.7	**86.1** ± 0.9
	best F1	85.6	86.6	87.6	**89**	87.2
Adrenal glands	Avg F1	70.6 ± 6.4	**74.5** ± 3.6	73.2 ± 4.6	71 ± 4.9	72.8 ± 1.9
	Best F1	79.7	77.4	**79.8**	76.5	76.1

In all the experiments presented in here, we used LoRA to train the model. For the lung experiments, we used 20% of the training data. Bold values show the best achieved value for specific metrics and specific organ data.

**Figure 5:**
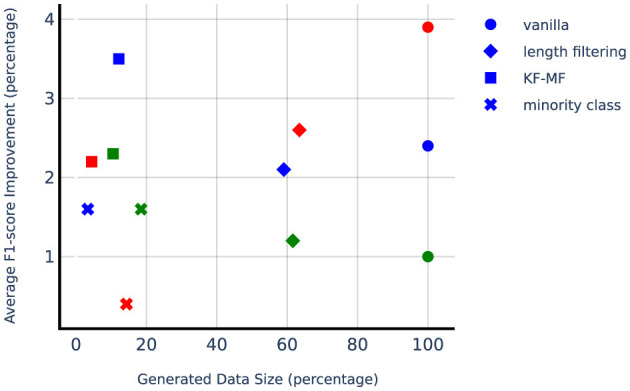
Average F1-score improvement from different augmentation techniques. Liver, lung, and adrenal glands are depicted with blue, green, and red markers, respectively. 100% of *Generated Data Size* refers to the number of synthetically generated samples when using vanilla data augmentation with *N* = 10, which is ten times the original training set size.

Figure 5 shows the improvement from DA methods compared to the no-augmentation baseline with respect to a number of synthetically generated samples, which is a good proxy for computational complexity since LLM size is orders of magnitude larger than SLM. Considering the computational complexity, KF-MF method is performing very impressively while adding a small amount of synthetically generated samples. Length filtering uses more than half of the generated samples and is more computationally expensive compared to the other targeted DA methods. Lung seems to benefit the most from minority class augmentation, which also has a higher relative number of generated samples compared to minority class augmentation on liver and adrenal glands. The liver dataset is not highly imbalanced, so the number of needed positive samples to balance the classes is small. Adrenal glands, on the other hand, have a small positive sample set, and even with the high upsampling factor, the absolute number of generated samples remains small.

### 4.2 Impact of report structure on performance

As shown in Table 4, the F1-score consistently improves with access to the structured findings section compared to using only the impression section, regardless of the organ or fine-tuning method. However, when patient history is available, the lung dataset does not show improvement from accessing the organ-specific subsection. This does not imply that the findings section lacks additional information but rather shows the model could not leverage that information for more accurate metastasis predictions. Both liver and adrenal glands datasets still benefit from report structure in multiple report settings. Nevertheless, access to historical data reduces the performance gap in terms of the average F1-score between the structured and non-structured report setting, from 7.8 to 6.7 on average across organs and fine-tuning methods. This effect is particularly notable when using LoRA for tuning, where the gap decreases from 7.3 to 4.5 points.

**Table 4:** Entries for avg F1-score rows are in the format of *average F1-score*±*standard deviation of F1-score*.

**Dataset**	**Metric**	**Findings + Impression**						**Impression**					
		***Single***			***Multi***			***Single***			***Multi***		
		**FFT**	**PT**	**LoRA**	**FFT**	**PT**	**LoRA**	**FFT**	**PT**	**LoRA**	**FFT**	**PT**	**LoRA**
Lung	Avg-F1	**78.4** ± 1.2	76.0 ± 1.3	77.1 ± 2	78.0 ± 2.7	75.7 ± 2.7	**78.9** ± 2.4	**71.4** ± 1.5	69.5 ± 1.4	69.7 ± 1.2	81.5 ± 1.3	76.4 ± 1.2	79.5 ± 1.5
	Best-F1	80.2	78.1	**80.4**	80.9	78.4	83.1	**73.9**	71.1	71.7	83.1	77.7	81.4
Liver	Avg-F1	**80.3** ± 1.1	79.5 ± 1.1	79.0 ± 0.6	83.9 ± 1.3	85 ± 1.6	91.2 ± 1.2	68.5 ± 0.7	68.6 ± 0.7	**69.7** ± 1.6	75.2 ± 0.9	77.9 ± 2.5	**82.6** ± 2
	Best-F1	**82.2**	80.7	79.9	85.1	86.8	92.9	69.7	69.5	**71.4**	76.7	80.8	**85.6**
Adrenal glands	Avg-F1	**68.0** ± 1.0	**68.0** ± 1.5	66.3 ± 1.6	71.1 ± 3.9	75.5 ± 1.8	71.0 ± 5.9	61.0 ± 1.4	**62.6** ± 0.9	61.1 ± 1.7	63.9 ± 2.5	67.1 ± 2.3	**70.6** ± 6.4
	Best-F1	69.5	**70.6**	67.7	75.7	78.2	80.0	62.5	**63.3**	**63.3**	66.7	71.1	**79.7**

The highest F1-score on each row is underlined. PT stands for prompt-tuning. Bold values are the best achieved value for a specific metrics and specific organ data under a certain data availability (for example single impression only).

### 4.3 Impact of prior reports on detection efficacy

In our previous work (Barabadi et al., 2024), we examined the effect of using patient history for metastases detection. Here, we conduct more extensive experiments, comparing the impact of utilizing past patient reports with various fine-tuning strategies and access levels. When structured findings are available, we observe average F1-score improvements of +2.1, +4.2, and +6.2 points with fine-tuning, prompt-tuning, and LoRA, respectively. With only the impression available, the improvements are +6.6, +6.9, and +10.7 points, respectively. While access to previous reports is always beneficial, the improvement is more pronounced when only the impression section is available. This suggests that patient history provides the model with additional crucial information for metastasis detection when the data from each visit is limited to the impression section.

### 4.4 Comparing PEFT methods

We utilize three different strategies to adapt the base model to the target task: vanilla fine-tuning, deep prompt-tuning, and LoRA. In general, PEFT methods performed better than vanilla fine-tuning with two exceptions: First, on the lung data set, vanilla fine-tuning is performing better or comparable with PEFT method across settings, which is probably because of the size of the dataset. PEFT methods proved more effective on our smaller data on liver and adrenal glands. Secondly, on structured report/single setup for the liver, fine-tuning outperforms PEFT methods by a small margin. In general, PEFT methods perform better than vanilla fine-tuning in terms of average and best F1-score on test sets. In particular, LoRA outperforms FFT by a large margin in multiple report settings.

## 5 Discussions

### 5.1 Study the diversity of generated examples

One of the objectives of DA is to increase the diversity of training samples, allowing the model to learn from examples that being presented in different forms and terminologies while conveying the same meaning. To quantify the diversity of synthetically-generated samples, we use self-BLEU score (Zhu et al., 2018), which has repurposed the original BLEU score (Papineni et al., 2002) for assessing the diversity of synthetically-generated text. The self-BLEU score calculates the lexical similarity by counting the number of shared n-grams between a list of references and a candidate up to *n*_*max*_, which has been set to 5. We measure self-BLEU of a generated candidate with respect to the original sample (single-reference) or the pool of generated samples from the same original sample (multi-reference). A lower self-BLEU score indicates higher lexical diversity.

While higher diversity is desirable, it is also important to preserve semantic information. To assess the semantic similarity between the generated samples and the original sample, we use the sentence-Transformers Python package to map the samples to a semantic space and measure the cosine similarity between the original and generated samples. Specifically, we use the paraphrase-distilroberta-base model, which is trained on paired paraphrased sentences. We present the diversity and similarity measurements in Table 5. The low self-BLEU single-reference scores indicate that the generated samples are lexically different from the original sample. However, the multi-reference self-BLEU scores are high, suggesting limited diversity among generated samples. It is important to note that as the number of samples being compared increases, the self-BLEU score will inevitably rise due to the limited ways of rewriting a sentence. High cosine similarity measurements indicate that our DA methods maintain high semantic fidelity.

**Table 5:** Lexical diversity and semantic fidelity measurement of synthetically generated samples.

	**Self-BLUE single reference**	**Self-BLUE multi reference**	**Cosine similarity**
Lung	0.13	0.85	0.94
Liver	0.12	0.88	0.93
Adrenal glands	0.14	0.86	0.95

The self-BLEU score ranges from 0 to 1, and a lower score represents higher diversity. Cosine similarity ranges from –1 to 1, and a higher score means higher semantic similarity.

### 5.2 Effective history duration in metastases analysis

**Figure 6:**
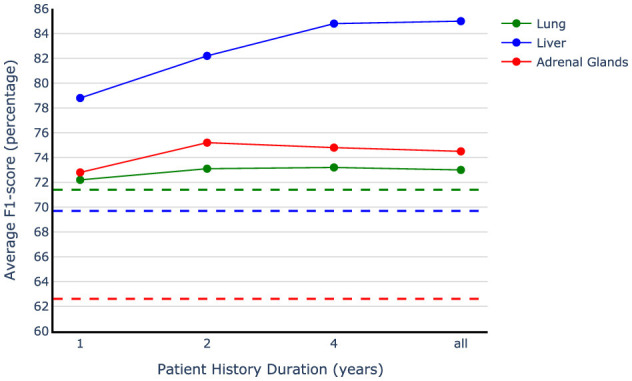
Patient history duration effect on model performance. The dotted lines show the best single report baselines in the impression-only setting for each organ. The models trained on the vanilla augmented data have been used to examine different history lengths.

The results presented in Table 4 clearly show the importance of including patient history in accurate metastases annotation. However, it is still unclear how far back in patient history we should look to accurately identify metastases. To gain a more nuanced understanding of the impact of patient history, we assess our method's performance by including only one, two, or five years of patient history in the testing phase. This allows us to observe how the models perform when given access to varying lengths of historical data. Figure 6 demonstrates our observations and indicates that the most recent data bring the most benefit to the model performance. We used the models trained on the vanilla augmented train sets and limited the history information during evaluation. Our observation shows more than eight points of improvement in the average F1-score by adding only the most recent year history, which includes three reports on average, compared to the best-performing single report baseline. The only exception is in the case of lung data; since the baseline for the lung is trained on the full dataset and the model used for history examination is trained only on a subset of data (20% of the original training set), they are not directly comparable. Our findings indicate that the exams conducted in the year leading up to the target examination contain crucial information for accurately interpreting the current report when only the impression section is available. Adding the second last year of history is helpful in improving the model performance by a smaller amount compared to the first year, and jumping to the five-year history setting results in a better F1-score for the liver organ, while for the other two organs, the performance starts saturating at two years point. Overall, we observe that two years of patient history is valuable to consider while performing metastases annotation, with the first year being the most beneficial.

## 6 Conclusion

This study presents an automated method for annotating radiology reports to identify metastatic sites in cancer patients by using free-text radiology reports. We show how Llama3, a recent open-source instruction-tuned LLM, can be prompted to generate high-quality clinical synthetic data and how training an SLM on that data enables transferring the LLM's extensive knowledge to a SLM while maintaining low deployment costs. Our approach effectively mitigates the labeled data scarcity issue in the clinical context. Furthermore, we introduce targeted data augmentation techniques which reduce synthetic data generation costs by selectively replicating more informative samples. Although introducing a more fine-grained structure to the radiology reports offers an advantage in metastasis identification, our results suggest that comparable performances can be achieved on non-structured reports by judiciously utilizing historical patient information. Our results showcase the effectiveness of data augmentation and utilizing patient history on liver, lung, and adrenal glands organs under different circumstances. Our work contributes to the growing body of research aimed at automating the analysis of clinical notes using NLP techniques, offering a broadly applicable low-cost solution to the task of metastases detection, which paves the way for a deeper analytical understanding of cancer progression patterns.

## Data Availability

The data analyzed in this study is subject to the following licenses/restrictions: HIPAA (Health Insurance Portability and Accountability Act) protection of personal health information. Requests to access these datasets should be directed to: dok@mskcc.org.

## Footnotes

^1^ https://www.who.int/health-topics/cancer

^2^ https://openai.com/index/chatgpt/
